# Jules Tinel (1879–1952)

**DOI:** 10.1007/s00415-015-7998-7

**Published:** 2016-01-02

**Authors:** Krzysztof Pietrzak, Andrzej Grzybowski, Jacek Kaczmarczyk

**Affiliations:** Department of Orthopaedics and Traumatology, University of Medical Sciences, 28 Czerwca 1956 135/147, 61-545 Poznań, Poland; Department of Ophthalmology, Poznań City Hospital, Poznań, Poland; Department of Ophthalmology, University of Warmia and Mazury, Olsztyn, Poland

Jules Tinel was born on 13 October 1879 in Rouen in France to a family with many generations of surgeons and doctors. He showed both scientific and sports talents from early childhood. He organised sports teams and showed an interest in painting and literature, and even contributed to local literary magazines. His other artistic attempts involved writing music. Tinel started his education in a catholic school, and then he started his studies for a degree in medicine at the University of Rouen. From 1900, he studied medicine in Paris, where he was awarded the degree of *externe des hôpitaux,* the first degree in medical education. He completed his studies in 1906. Influenced by one of his tutors, Joseph Jules Dejerine (1849–1917), he started to specialise in neurology. In 1910, he received a doctorate for his work on neurological consequences of syphilis [1]. In 1911, Tinel became head of the Pitié-Salpêtrière Hospital in Paris and 2 years later also the head of the hospital laboratory. In 1914, Tinel was accepted as a member of the Société de Neurologie and in 1936 he became the president of the institution.

In 1914, Tinel was appointed head doctor of the neurology unit in a hospital in Le Mans. After the First World War broke out, he was mobilised and became an assistant doctor in an infantry regiment. It was at that time that he became interested in consequences of damage to peripheral nerves resulting from ballistic trauma. At that time doctors had already been aware of sensation disorders and paresthesias related to nerve damage, yet these were not considered to be of any clinical or diagnostic significance [2]. Tinel’s detailed research resulted in a description of an effect where tapping of the damaged nerve generated paresthesis (tingling sensation). He also discovered that progressing regeneration of the damaged nerve, still without myelin sheath, gives a more peripheral occurrence of the sign [3]. This sign was named after Tinel. His next work was based on the observations of 693 patients [4]. In the decades to follow the sign was observed also in nerve compression syndromes, as for example in carpal tunnel syndrome [5].

The sign attributed to Tinel had been described by German doctor, Paul Hoffmann (1884–1962) several months before Tinel did. Hoffmann was a doctor on the other side of the frontline. His first publication was dated March 1915, the next one came out several months later [6, 7]. Because of the information blockade neither author was aware of the other’s papers. The news of Hoffman’s discovery failed to reach a wider audience due to a lower rank of the periodical and censorship affecting publications from the Central Powers. His contribution to the discovery was neglected throughout the first half of the 20th century.

Undoubtedly, contrary to Hoffmann’s work, Tinel’s descriptions are much more detailed and insightful. He clearly separated pain from tingling, and found that the latter had a high prognostic value, and was a sign that the nerve was regenerating. He also found that if the tingling no longer progressed, a surgical intervention was required as it was a signal of a mechanical obstacle to nerve regeneration. Tinel also linked sensation disorders and nerve regeneration with the presence of neuroma. He described not only the regeneration of sensory nerves, but also accurately predicted that tingling was also a sign that motor nerves would regenerate [3, 4]. The name predominantly used in pertinent literature nowadays is Tinel-Hoffmann’s sign, which seems to be the most accurate.

After the War ended and Tinel was demobilised, he focused his efforts on psychosomatic diseases and symptoms of encephalitis. In 1922, Tinel and his team were the first to describe paroxysmal hypertension in pheochromocytoma [8]. From 1922 to 1936 he worked at La Rochefoucauld, where he managed to set up a research laboratory despite insufficient facilities. He concentrated on research into measurements of intracranial pressure and studies of the autonomic system [9], the impact of histamines on blood vessels and regulation of blood circulation in the brain [10]. From 1936 Tinel worked in Clichy and in 1940 he returned to Paris.

During the Second World War Tinel was involved in the French resistance movement. He organised a network for transporting to Spain wounded pilots whose planes were shot down over France. His son Jacques Tinel, who was actively involved in these actions, was arrested and sent to the concentration camp in Mittlebau-Dora, where he died. Tinel himself, together with his wife and the other son, were arrested in France and released from prison after a few months. In 1945, Tinel retired, but continued his work in Paris. In 1947, he suffered from cerebral ischemia and aphasia, but he recovered. He died of heart failure on 4 March 1952.
